# Optimized gold nanoshell ensembles for biomedical applications

**DOI:** 10.1186/1556-276X-8-142

**Published:** 2013-03-28

**Authors:** Debabrata Sikdar, Ivan D Rukhlenko, Wenlong Cheng, Malin Premaratne

**Affiliations:** 1 Advanced Computing and Simulation Laboratory (A χL), Department of Electrical and Computer Systems Engineering, Monash University, Clayton 3800, Victoria, Australia; 2 Department of Chemical Engineering, Faculty of Engineering, Monash University, Clayton 3800, Victoria, Australia; 3, The Melbourne Centre for Nanofabrication, 151 Wellington RoadClayton 3168, Victoria, Australia

**Keywords:** Hollow gold nanoshells, Lognormal distribution, Absorption, Scattering, Biomedical applications

## Abstract

We theoretically study the properties of the optimal size distribution in the ensemble of hollow gold nanoshells (HGNs) that exhibits the best performance at *in vivo* biomedical applications. For the first time, to the best of our knowledge, we analyze the dependence of the optimal geometric means of the nanoshells’ thicknesses and core radii on the excitation wavelength and the type of human tissue, while assuming lognormal fit to the size distribution in a real HGN ensemble. Regardless of the tissue type, short-wavelength, near-infrared lasers are found to be the most effective in both absorption- and scattering-based applications. We derive approximate analytical expressions enabling one to readily estimate the parameters of optimal distribution for which an HGN ensemble exhibits the maximum efficiency of absorption or scattering inside a human tissue irradiated by a near-infrared laser.

## Background

The biocompatibility of gold nanoparticles, along with their tunable plasmon resonances and the ability to accumulate at targeted cancer sites, has proven them to be very effective agents for absorption-based photothermal therapy and scattering-based imaging applications [1-8]. Amongst the commonly used gold nanoparticles, silica-core gold nanoshells exhibit larger photothermal efficiency as compared to gold nanorods of equal number densities [1], whereas hollow gold nanoshells (HGNs) absorb light stronger than the silica-core gold nanoshells do [9,10]. Furthermore, HGNs are comparatively less harmful to healthy tissues neighboring a cancer site [9], which makes them prospective for both photothermal and imaging applications. Although different tissue types and excitation wavelengths were analyzed before to determine the optimal dimensions of a nanoshell [10,11], no optimization has ever been performed for a nanoshell ensemble with a real size distribution. In this Letter, we fill this gap by conducting the first theoretical study of the distribution parameters of the lognormally dispersed HGNs exhibiting peak absorption or scattering efficiency. In particular, we comprehensively analyze the dependence of these parameters on the excitation wavelength and optical properties of the tissue, giving clear design guidelines.

## Methods

Despite a significant progress in nanofabrication technology over the past decade, we are still unable to synthesize large ensembles of almost identical nanoparticles. The nanoparticle ensembles that are currently used for biomedical applications exhibit broad size distributions, which are typically lognormal in shape [12-15]. In an ensemble of single-core nanoshells, both the core radius *R* and the shell thickness *H* are distributed lognormally [15], with their occurrence probabilities given by the function [16]

(1) $$f(x;\mu_{X},\sigma_{X})=\frac{1}{x\sigma_{X}\sqrt{2\Pi }}\;\exp\left(-\;\frac{{\left(\ln x-\mu_{X}\right)}^{2}}{2\overset{2}{\underset{X}{\sigma }}}\right),$$

where *x*=*r* or *h* is the radius or thickness of the nanoshell, *μ*_*X*_= ln(Med[*X*]) and *σ*_*X*_ are the mean and standard deviation of ln*X*, respectively, and Med[*X*] is the geometric mean of the random variable *x*=*r* or *H*.

The efficiencies of absorption and scattering by a nanoparticle ensemble are the key characteristics determining its performance in biomedical applications. In estimating these characteristics, it is common to use a number of simplifying assumptions. First of all, owing to a relatively large interparticle distance inside human tissue (typically constituting several micrometers [17]), one may safely neglect the nanoparticle interaction and the effects of multiple scattering at them [18,19]. Since plasmonic nanoparticles can be excited resonantly with low-intensity optical sources, it is also reasonable to ignore the nonlinear effects and dipole–dipole interaction between biomolecules [20]. The absorption of the excitation light inside human tissue occurs on a typical length scale of several centimeters, within the near-infrared transparency window of 650 to 1000 nm [21]. However, the attenuation of light does not affect the efficiencies of scattering and absorption by the ensemble, and is therefore neglected in the following analysis. These simplifications allow us to relate the average absorption and scattering efficiencies (*S*_abs_ and *S*_sca_) of the nanoshell ensemble embedded in a tissue to the corresponding efficiencies (*Q*_abs_ and *Q*_sca_) of individual plasmonic nanoshells as

(2) $$S_{\alpha }=\iint Q_{\alpha }(r,h)\;f(r;\mu_{R},\sigma_{R})f(h;\mu_{H},\sigma_{H})\;dr\;dh,$$

where *Q*_*α*_(*r*,*h*) is expressed through Mie coefficients for a coated sphere [9,22,23], which are the functions of the excitation wavelength, refractive index of the tissue, and permittivities of the nanoshell constituents.

It is seen that the average absorption and scattering efficiencies of a nanoshell ensemble, excited at a fixed wavelength, are functions of the four parameters: Med[*R*], Med[*H*], *σ*_*R*_, and *σ*_*H*_. This poses the problem of finding, and studying the properties of, the optimal distribution parameters for which the nanoshell ensemble exhibits the maximum absorption or scattering efficiency.

## Results and discussions

**Figure 1:**
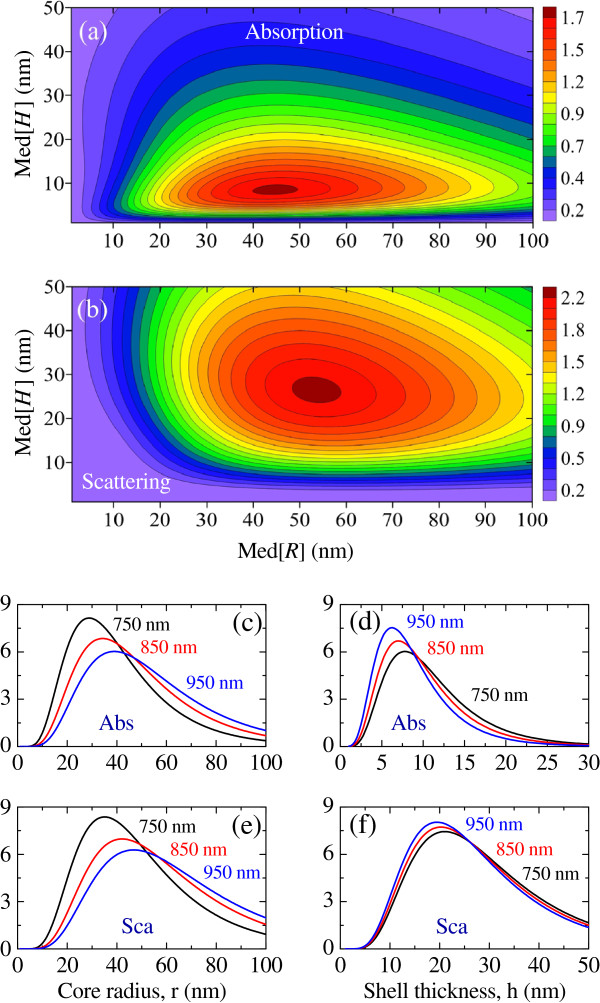
**Average (a) absorption and (b) scattering efficiencies of an hollow-gold-nanoshell ensemble with lognormal distribution.** The ensemble is excited by monochromatic light at *λ*=850 nm. Optimal distributions of core radius and shell thickness for maximum [(**c**) and (**d**)] absorption and [(**e**) and (**f**)] scattering efficiencies of the ensemble excited at *λ*=750, 850, and 950 nm. In all cases, *n*=1.55 and *σ*_*R*_=*σ*_*H*_=0.5.

We focus on HGNs with gold permittivity described by the size-dependent model from Ref. [9], and begin by evaluating their average absorption and scattering efficiencies inside a tissue of refractive index *n*=1.55. Figures 1(a) and 1(b) show these efficiencies in the parametric space of Med[*R*] and Med[*H*] for *σ*_*R*_=*σ*_*H*_=0.5 and excitation wavelength *λ*=850 nm. Each dependency is seen to exhibit a distinct peak in the form of a flat plateau, which arise predominantly due to the resonant interaction of light with the localized symmetric plasmon modes of the HGNs [9]. The absorption peaks for Med[*R*]≈44 nm and Med[*H*]≈9 nm, while the scattering reaches its maximum for larger and much thicker nanoshells, with Med[*R*]≈54 nm and Med[*H*]≈26 nm. The broadness of the peaks and the associated high tolerance of the nanoshell ensemble to the fabrication inaccuracies are the consequences of size distribution.

The effects of the excitation wavelength on the optimal distributions of the core radius and shell thickness are shown in Figures 1(c)– 1(f). Equal *σ*_*R*_ and *σ*_*H*_ (*σ*_*R*_=*σ*_*H*_=*σ*) correspond to the situation of similar (scalable) shapes of the two distributions. It is seen that the increase in the excitation wavelength shifts the optimal distribution *f*(*r*;*μ*_*R*_,*σ*) towards larger radii for both absorption [Figure 1(c)] and scattering [Figure 1(e)]. This trend is opposite to the behavior of the optimal distributions *f*(*h*;*μ*_*H*_,*σ*) in Figures 1(d) and 1(f), which shifts towards thinner shells with *λ*. Since the increase in Med[*R*] is larger than the reduction in Med[*H*], the optimal excitation of ensembles with larger HGNs require lower-frequency sources.

**Figure 2:**
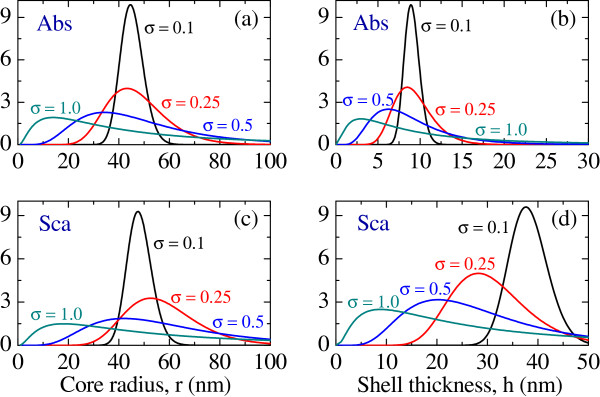
**Optimal lognormal distributions of core radius and shell thickness in an ensemble of hollow gold nanoshells exhibiting maximum average [(a) and (b)] absorption and [(c) and (d)] scattering efficiencies for*σ=σ*R*=σ*H*=0.1*, 0.25, 0.5, and 1.0.** The simulation parameters are the same as in Figures 1(a) and 1(b).

The optimal geometric means of HGNs’ dimensions crucially depend on the shape of size distribution determined by the parameter *σ*. Figure 2 shows how the optimal distributions of *R* and *H* are transformed when *σ* is increased from 0.1 to 1. As expected, larger *σ* results in broader distributions that maximize the absorption and scattering efficiencies of the nanoshell ensemble. It also leads to the right skewness of the distributions, thus increasing the fabrication tolerance. At the same time, the increase in *σ* from 0.1 to 1 reduces the peak values of *S*_abs_ and *S*_sca_ by about a factor of 3.5 each. This indicates the need of a compromise between the performance of an HGN ensemble and the fabrication tolerance. Regardless of *σ*, the ensemble exhibiting the maximum absorption efficiency comprises of HGNs with core radii smaller than those required for maximizing the scattering efficiency. A similar trend exists for the optimal distribution *f*(*h*;*μ*_*H*_,*σ*), with absorbing nanoshells being much thinner than the scattering ones.

**Figure 3:**
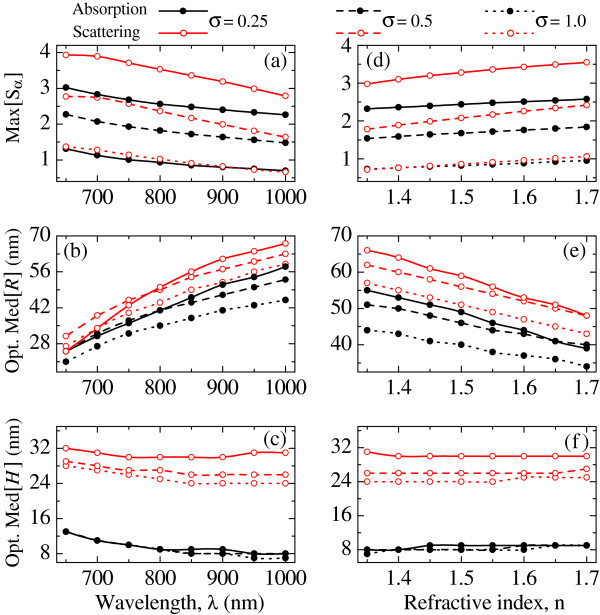
**[(a) and (d)] Optimal average absorption (filled circles) and scattering (open circles) efficiencies, and parameters [(b) and (e)]*Med[R]* and [(c) and (f)]*Med[H]* of the corresponding optimal distributions as functions of excitation wavelength and tissue refractive index.** In (**a**)–(**c**), *n*=1.55; in (**d**)–(**f**), *λ*=850 nm. Solid, dashed, and dotted curves correspond to *σ*=0.25, 0.5, and 1.0, respectively.

The dependencies of the peak absorption and scattering efficiencies on the excitation wavelength are plotted in Figure 3(a) for *n*=1.55. The efficiencies are seen to monotonously decrease with *λ*, which makes shorter-wavelength near-infrared lasers preferable for both absorption- and scattering-based applications. Figures 3(b) and 3(c) show the dispersion of the geometric means for the optimal nanoshell distributions. One can see that the best performance is achieved for the nanoshells of smaller sizes, excited at shorter wavelengths. These results are summarized in the following polynomial fittings of the theoretical curves: Med[*R*]≈*λ*(21*σ*^2^−61*σ*+106)−44*σ*^2^+72*σ*−48 and Med[*H*]≈*λ*^2^(−58*σ*^2^+65*σ*+44)+*λ*(103*σ*^2^−127*σ*−78)−56*σ*^2^+77*σ*+39 for absorption, and Med[*R*]≈*λ*(281*σ*^2^−409*σ*+225)−266*σ*^2^+376*σ*−146 and Med[*H*]≈*λ*^2^(−966*σ*^3^+1921*σ*^2^−1150*σ*+244)+*λ*(1731*σ*^3^−3439*σ*^2^+2046*σ*−430)−803*σ*^3^+1607*σ*^2^−967*σ*+231for scattering. Here *λ* is expressed in micrometers, 0.1≤*σ*≤1, and the accuracy of the geometric means is about ±1 nm.

The parameters of the optimal lognormal distribution also vary with the type of human tissue. Figures 3(d)–3(f) show such variation for the entire span of refractive indices of human cancerous tissue [9,19], *λ*=850 nm, and three typical shapes of the distribution. It is seen that the peak efficiencies of absorption and scattering by an HGN ensemble grow with *n* regardless of the shape parameter *σ*. The corresponding geometric mean of the core radii reduces with *n* and may be approximated as Med[*R*]≈*n*(−51*σ*^2^+87*σ*−65)+72*σ*^2^−136*σ*+147 for absorption, and as Med[*R*]≈*n*(−94*σ*^2^+142*σ*−87)+114*σ*^2^−179*σ*+178 for scattering. In contrast, the optimal geometric mean of the shell thicknesses is almost independent of *n* and approximated by the polynomial Med[*H*]≈2*σ*^2^−3*σ*+10 in the case of absorption, and as Med[*H*]≈26*σ*^2^−41*σ*+40 in the case of scattering. These expressions allow estimation (with an accuracy of about ±1 nm) of the optimal distribution parameters of an HGN ensemble excited at *λ*=850 nm for 0.1≤*σ*≤1 and 1.35≤*n*≤1.7. Numerical calculations show that the optimal dependencies Med[*R*](*n*) and Med[*H*](*n*) have almost constant slopes for 650 nm≤*λ*≤1000 nm. This feature allows one to use Figure 3 to roughly estimate the optimal lognormal distributions of HGNs to be delivered to any human tissue illuminated by a near-infrared laser.

## Conclusions

In summary, we have studied the optimal distributions of lognormally dispersed hollow gold nanoshells for different excitation wavelengths and human tissues. Shorter-wavelength, near-infrared sources were found to be most effective for *in vivo* biomedical applications. The analytical expressions obtained may be used to estimate the optimal distribution of the nanoshells providing the maximum efficiency of their absorption or scattering of near-infrared radiation inside human tissue.

## Abbreviations

HGN: Hollow Gold Nanoshell; Med[ *X* ]: Median of the random variable *X*.

## Competing interests

The authors declare that they have no competing interests.

## Authors’ contributions

IDR, WC, and MP jointly suggested the study conducted by DS. DS conceived the model, performed the simulations, and prepared the manuscript. IDR, WC, and MP supervised the study, participated in the analysis of the results, helped DS to interpret and present the obtained result, and thoroughly edited the manuscript. All authors read and approved the final manuscript.

## References

[B1] Pattani VP Tunnell JW Nanoparticle-mediated photothermal therapy: A comparative study of heating for different particle types Lasers Surg Med 2012 8 675—684 22933382 10.1002/lsm.22072 PMC3512106

[B2] Akiyama Y Mori T Katayama Y Niidome T Conversion of rod-shaped gold nanoparticles to spherical forms and their effect on biodistribution in tumor-bearing mice Nanoscale Res Lett 2012 8 565 10.1186/1556-276X-7-565 23050635 PMC3492114

[B3] Kennedy LC Bear AS Young JK Lewinski NA Kim J Foster AE Drezek RA T cells enhance gold nanoparticle delivery to tumors in vivo Nanoscale Res Lett 2011 8 283 10.1186/1556-276X-6-283 21711861 PMC3211348

[B4] Huang X El-Sayed MA Plasmonic photo-thermal therapy (PPTT) Alex J Med 2011 8 1 9 10.1016/j.ajme.2011.01.001

[B5] Liu L Guo Z Xu L Xu R Lu X Facile purification of colloidal NIR-responsive gold nanorods using ions assisted self-assembly Nanoscale Res Lett 2011 8 143 10.1186/1556-276X-6-143 21711657 PMC3211191

[B6] Verma VC Singh SK Solanki R Prakash S Biofabrication of anisotropic gold nanotriangles using extract of endophytic Aspergillus clavatus as a dual functional reductant and stabilizer Nanoscale Res Lett 2011 8 16 10.1007/s11671-010-9743-6 PMC3211211 27502640

[B7] Chen Y Hung Y Liau I Huang GS Assessment of the in vivo toxicity of gold nanoparticles Nanoscale Res Lett 2009 8 858 864 10.1007/s11671-009-9334-6 20596373 PMC2894102

[B8] Jain PK Lee KS El-Sayed IH El-Sayed MA Calculated absorption and scattering properties of gold nanoparticles of different size, shape, and composition: Applications in biological imaging and biomedicine J Phys Chem B 2006 8 7238 7248 10.1021/jp057170o 16599493

[B9] Sikdar D Rukhlenko ID Cheng W Premaratne M Effect of number density on optimal design of gold nanoshells for plasmonic photothermal therapy Biom Opt Express 2013 8 15 31 10.1364/BOE.4.000015 PMC3539187 23304644

[B10] Kessentini S Barchiesi D Quantitative comparison of optimized nanorods, nanoshells and hollow nanospheres for photothermal therapy Biom Opt Express 2012 8 590 604 10.1364/BOE.3.000590 PMC3296544 22435104

[B11] Grosges T Barchiesi D Kessentini S Grehan G de la Chapelle ML Nanoshells for photothermal therapy: A Monte-Carlo based numerical study of their design tolerance Biom Opt Express 2011 8 1584 1596 10.1364/BOE.2.001584 PMC3114226 21698021

[B12] López-Muñoz GA Pescador-Rojas JA Ortega-Lopez J Salazar JS Balderas-López JA Thermal diffusivity measurement of spherical gold nanofluids of different sizes/concentrations Nanoscale Res Lett 2012 8 423 10.1186/1556-276X-7-423 22846704 PMC3478211

[B13] Tengen TB Designing nanomaterials with desired mechanical properties by constraining the evolution of their grain shapes Nanoscale Res Lett 2011 8 585 10.1186/1556-276X-6-585 22067060 PMC3314297

[B14] Amendola V Meneghetti M Size evaluation of gold nanoparticles by UV vis spectroscopy J Phys Chem C 2009 8 4277 4285 10.1021/jp8082425

[B15] Wu G Mikhailovsky A A KH A ZJ Synthesis, characterization, and optical response of gold nanoshells used to trigger release from liposomes Methods Enzymology 2009 8 279 307 10.1016/S0076-6879(09)64014-3 19903560

[B16] Crow EL Shimizu K Lognormal distributions: Theory and applications 1988 New York: M. Dekker

[B17] Kah JCY Chow TH Ng BK Razul SG Olivo M Sheppard CJR Concentration dependence of gold nanoshells on the enhancement of optical coherence tomography images: a quantitative study Appl Opt 2009 8 D96 D108 10.1364/AO.48.000D96 19340129

[B18] Handapangoda CC Premaratne M Paganin DM Hendahewa PRDS Technique for handling wave propagation specific effects in biological tissue: Mapping of the photon transport equation to maxwell’s equations Opt Express 2008 8 17792 17807 10.1364/OE.16.017792 18958061

[B19] Vo-Dinh T Biomedical Photonics Handbook 2003 CRC, Boca Raton: Florida

[B20] Rubinov AN Afanas’ev AA Nonresonance mechanisms of biological effects of coherent and incoherent light Opt Spectrosc 2005 8 943 948 10.1134/1.1953991

[B21] Yu G Near-infrared diffuse correlation spectroscopy in cancer diagnosis and therapy monitoring J Biom Opt 2012 8 010901 010911 10.1117/1.JBO.17.1.010901 PMC3380819 22352633

[B22] Tang Y Vlahovic B Metallic nano-particles for trapping light Nanoscale Res Lett 2013 8 65 10.1186/1556-276X-8-65 23391493 PMC3576255

[B23] Bohren CF Huffman DR Absorption and scattering of light by small particles 1998 New York: Wiley

